# 

**Published:** 1913-05-24

**Authors:** 


### Public Pharmacists' and Dispensers' Association.

At the fifteenth annual Bohemian concert of this Association, Mr. R. W. Lindsey, M.P.S. (chairman of the Association), took the chair. Mr. G. W. Gibson, M.P.S., was vice-chairman, and there was a representative gathering, including Mr. C. B. Allen (president of the Pharmaceutical Society). Mr. T. H. W. Idris, J.P. (president of the Association), Mr. J. H. France (secretary), Mr. R. R. Bennett (University Hospital), Colonel Probyn, Dr. W. H. France, Dr. Coulson, Dr. Churchill Sibley, Mr. Herbert Antcliffe (Sheffield), Mr. F. N. C. Clark, and Mr. G. W. Lindsay were also present.

Mr. T. H. W. Idris, J.P., who has been president of the Association for some ten or twelve years, spoke of the good work the Association is doing, and commended Messrs. Lindsey, France, and Gibson for the excellent work they had done on the Association's behalf.

The President, Mr. C. B. Allen, who very kindly attended (although having spent a full day at other meetings), made a presentation of Ruskin's Works to Mr. W. E. Miller, M.P.S., "a veteran," on behalf of the members. Mr. Allen spoke of his personal knowledge of Mr. Miller's studious nature, his frequent visits to the Society's library, and attendance at the meetings of that body, and wished him a happy retirement.

Mr. Miller, who has been in the service of the St. Pancras Guardians since 1877, replied, and gave some of his early experiences in the Poor Law. Mr. Miller intends to retire shortly on a well-earned pension.

Mr. G. W. Gibson proposed the toast of the Chairman, and mentioned the appointment of Mr. Lindsey on the Pharmaceutical Standing Committee on Insurance as a great honour for the Association. The musical programme was all that could be desired, and did great credit to the concert committee.

### Personalia.

### The Late Sir E. Hay Currie.

At a largely attended meeting of the Council of the Hospital Sunday Fund on the 19th inst., Sir John Bell in the chair, it was proposed by Sir Ernest Tritton, Bart., seconded by Sir Richard B. Martin, Bart., and unanimously resolved, that: The Council has received with very great regret the news of the death of Sir Edmund Hay Currie and desires to place on record its high appreciation of his services on behalf of the Fund. From its foundation in 1872 till 1902 Sir Edmund Hay Currie was one of its honorary secretaries, and in 1902 became its paid secretary, holding that post until his death. The Council will never forget how much of the success of the Fund has been due to his personality, and how for over forty years he devoted his life to increasing year by year the amount of the collections, thus rendering invaluable assistance to the hospitals and dispensaries of the Metropolis. The Council desires to express its deepest sympathy with Lady Currie and the family in the great loss that they have sustained, and to assure them that the name of Sir Edmund Hay Currie will always be most thankfully remembered in connection with the work and progress of the Fund.

### Medals for Pharmacists.

The Hanbury Medal of the Pharmaceutical Society, given biennially for excellence in original research in chemistry and the natural history of drugs, has been awarded to Dr. F. B. Power, Ph.D., the director of the Wellcome Chemical Research Laboratories. Mr. John C. Jinks has gained the Pereira medal, while the Society's silver and bronze medals have been won respectively by Mr. Sydney K. Crews and Mr. William Cooper.

Professor Sir T. Clifford Allbutt has been re-elected by the Senate a representative of Cambridge University on the General Medical Council for five years from May 28, 1913.

Mr. C. H. Garlend, who has been the chairman of the National Sanatorium Association since its inception, has resigned, in consequence of his appointment to an inspectorship under the National Health Insurance Act.

The result of the open competition for the proposed cottage hospital at Staines is the selection of a design by Mr. Leslie T. Moore, A.R.I.B.A. This is the fourth small hospital for which this architect's design has been accepted, the recently rebuilt cottage hospital at Axminster being among them.

The Acton Urban District Council for the third time in succession has reappointed Miss Councillor S. M. Smee as chairman of their public health and isolation hospital committee. In previous years of office as chairman she rendered service to the committee and to the nursing staff of the Council's isolation hospital, who found in her a champion in the attacks made upon the hospital staff, particularly a year or two back. "Her entry into the local government of Acton," writes a correspondent, "undoubtedly had a softening effect upon the bitterness which often characterised the Council's proceedings."

### Gifts and Bequests.

The Trustees of the late Sir Julius Wernher have voted £1,500 to the fund which Mr. Austen Chamberlain is raising for the extension of the London School of Tropical Medicine.

Mr. Isaac Danson, of Aston, Birmingham, has left £250 to Dr. Barnardo's Homes and £1,250 to endow a bed in the General Hospital, Birmingham, in memory of himself and his sister, Jane Danson.

Mr. Edward Beck, of Isleworth, has bequeathed, subject to a life interest, £8,000 to University College Hospital, London. He left the residue of his property for division into seventy-four parts, with four parts to the Warrington Infirmary.

St. Mary's Hospital, Paddington, and the Royal Dental Hospital, Leicester Square, have received £400 each from the Trustees of Smith's (Kensington Estate) Charity; the Mount Vernon Hospital for Consumption and Diseases of the Chest, Hampstead and Northwood, and the Royal London Ophthalmic Hospital £200 each; and the Infants Hospital, Vincent Square, Westminster, £50.

Mr. Abraham Haigh Crowther, flannel manufacturer, has bequeathed £5,000 to the Rochdale Infirmary and Dispensary to endow five beds there; £3,000 to the Queen Victoria Memorial Nurses' Home, Rochdale, for the endowment; and £200 each to the Rochdale and District Society for Visiting and Instructing the Blind, the Rochdale Adult Deaf and Dumb Society, and the Children's Convalescent Home, St. Anne's-on-Sea.

The Executors of the late Sir Julius C. Wernher, Bart., are now distributing the sums allocated by them to various charitable institutions out of the £100,000 bequeathed to them for this purpose. It is understood that, as a general rule, the Executors have not included, among the institutions benefited, any of the hospitals which receive support from King Edward's Hospital Fund, as the testator considered that these hospitals would participate in the large bequests made by him to that fund.

### IMPORTANT GIFT TO HORTON INFIRMARY, BANBURY.

Mr. Albert Brassey has given £500 to the Horton Infirmary, Banbury, to wipe out the debt, and to prevent, if possible, the sale by the trustees of five acres of land close to the hospital, against which there has been a strenuous opposition by many of the governors and the medical staff.

### Appointments.

Dr. Herbert Vachell has resigned his post as honorary physician to King Edward VII.'s Hospital, Cardiff, which he has held for twenty-eight years.

Mr. E. W. Hey Groves has been appointed honorary surgeon to the Bristol General Hospital, where he has done ten years' work as assistant surgeon. Mr. Hey Groves is an F.R.C.S. and a graduate of London University. His successor as assistant surgeon at the hospital is Mr. C. A. Moore, a former senior house surgeon.

### Books Received.

George Allen and Co.: "The Interpretation of Dreams," by Prof. Sigmund Freud, LL.D., translated by A. A. Brill, M.D.

W. H. and L. Collingridge: "City of London Year Book and Civic Directory, 1913."

H. K. Lewis: "The Mineral Waters of Vichy," by Charles Cotar.

L. N. Fowler and Co.: "Modern Miracles," by J. Wallace-Clarke.

Thacker, Spink and Co. (Calcutta): "The Indian Manual of First Aid," by Major R. J. Blackham.

E. Arnold: "Old Age: Its Care and Treatment," by Robert Saundby.

John Murray: "The Reduction of Domestic Flies," by E. H. Ross; "Researches into Induced Cell-Reproduction and Cancer," Vol. III.

Macmillan and Co.: "Babies," by Margaret French.

Clarendon Press: Oxford Medical Publications—"Laboratory Handbook of Bacteriology," by M. A. Gordon, M.A., M.D.; "The Practitioner's Encyclopædia," by J. Keogh Murphy.

A. and C. Black: "Text-Book of Midwifery," by R. W. Johnstone; "The Social Guide, 1913."

Baillière, Tindall and Cox: "The Nauheim Treatment of Diseases of the Heart," by Leslie Thorne Thorne.

Oliver and Boyd: "Food and Feeding in Health and Disease," by Chalmers Watson.

Eyre and Spottiswoode: "Printers' Pie."

The Scientific Press, Ltd.: "S.P." Pocket Guide Series—"Swedish Exercises," by T. D. Luke, M.D., F.R.C.S.; "Bandaging Made Easy," by Miss M. R. Hosking; "Essentials of Fever Nursing," by Lytton Maitland, M.D.; "How to Write and Read Prescriptions," by Lytton Maitland, M.D.; "Prevention of Consumption and Care of the Consumptive at Home," by Helen M. Brown.

### DOCTORS' WILLS.

Professor George Jordan Lloyd, M.D., M.S., M.Sc., F.R.C.S., of Edgbaston, has left estate valued at £60,213 gross, with net personalty £58,587.

Dr. Philip Frank, of South Kensington, who saw service with the Anglo-American Ambulance during the Franco-German War, and who died on March 17, aged eighty-two, left estate of the gross value of £70,429.

### Buildings Contemplated.

Abergavenny.—The Guardians have determined to purchase a site for a new infirmary.

Banffshire.—Plans have been approved by the County Council for alterations to Chalmer's Hospital, the cost of which is estimated at £600. Plans for alterations to Dufftown Isolation Hospital and Ladybrae Hospital, including a stone and lime block for the latter, have also been approved, the total cost being estimated at £2,910.

Bromley.—The Bromley and Beckenham Joint Hospital Board are negotiating for a loan of £5,273 for alterations and additions to the hospital.

Burton-on-Trent.—The final estimates for alteration and additions to the sanatorium, and to buildings in Union Street for treating tuberculosis, have just been completed, and amount to £2,520. This sum covers alterations to the dispensary, including furnishing and shelters, furnishing of sanatorium and administrative block, the extension of administrative block, and the addition of verandah and observation wards to the sanatorium.

Eastbourne.—The Guardians have adopted a scheme for certain additions to the infirmary.

Whitby.—It is reported that the Committee of the Cottage Hospital have under consideration an extension scheme estimated to cost from £800 to £900.

Winsley.—Wiltshire County Council have decided to apply to the Local Government Board for borrowing powers in reference to sanatorium accommodation.

